# Clinical efficacy of Shaomazhijing granules in the treatment of Tourette’s syndrome: a randomized controlled trial

**DOI:** 10.1186/s41065-025-00462-z

**Published:** 2025-05-31

**Authors:** Yan-Zhen Wang, Jiang Yang, Xin-Min Han

**Affiliations:** 1 Nanjing University of Chinese Medicine, Nanjing, Jiangsu 210023 China; 2 Department of Rehabilitation, Children’s Hospital of Shanxi, Taiyuan, Shanxi 030012 China; 3 Department of Pediatrics, Affiliated Hospital of Nanjing University of Chinese Medicine, Hanzhong Road, 155, Qinhuai District, Nanjing, Jiangsu 210029 China

**Keywords:** Chinese patent medicine, Shaomazhijing granules, Tiapride, Tourette’s syndrome

## Abstract

**Objective:**

We designed this study to verify the clinical efficacy and safety of Shaomazhijing granules, a Chinese patent medicine, in the treatment of Tourette's syndrome (TS) with liver-yang hyperactivity, liver wind, and phlegm-fire disturbance.

**Methods:**

We enrolled a total of 603 children and adolescents aged 5–18 years with TS in this randomized, double-blinded, multicenter study. We randomly assigned participants to a Shaomazhijing granules group, a Tiapride group, or a placebo group in a ratio of approximately 3:1:1, respectively. We evaluated the treatment results using the traditional Chinese medicine (TCM) syndrome quantitative classification scale and also compared the incidence of adverse events among the three groups.

**Results:**

The TCM syndrome of all patients improved over time. At week eight of TCM treatment, the overall syndrome score, primary symptoms (muscle tics), and secondary symptoms (emotional and psychological) of patients in the Shaomazhijing granules and tiapride groups showed significant improvements when compared to that of patients in the placebo group. Compared with the tiapride group, the Shaomazhijing granules group showed better improvement in the secondary symptoms (*P* < 0.05). While the clinical efficacy for primary symptoms of patients in the Shaomazhijing granules was similar (*P* = 0.969) with that of patients in tiapride groups. The TCM syndrome clinical control rate and the clinically excellent effectiveness rate of the Shaomazhijing granules group (3.45% and 44.51%) and tiapride group (2.86% and 26.67%) were higher than that of the patients in placebo group (1.04% and 12.50%, *P* < 0.001). Patients in placebo group (11.2%) and Shaomazhijing granules group (13.8%) had significantly lower overall adverse event rates in comparison with those in tiapride group (26.8%, *P* = 0.002).

**Conclusion:**

The clinical efficacy of Shaomazhijing granules is comparable to tiapride in reducing reducing the primary symptoms (muscle tics) of TS. Besides, it showed better efficacy in improving secondary (emotional and psychological) symptoms. Its safety profile is better than tiapride. Based on these results, Shaomazhijing granules can be considered a safe and effective treatment for patients with TS.

## Introduction

Tourette's syndrome (TS) is a common neuropsychiatric disorder that develops in childhood. It is characterized by involuntary, rigid, and rapid motor and/or vocal twitches in the muscles of the head, face, trunk, and limbs; among these, head and facial muscle twitches are the most common, accompanied by emotional and psychological changes [1, 2]. Because of its rising prevalence and uncertain pathophysiology, TS is a major area of focus for researchers interested in child mental health worldwide [3–5].

Currently, there are a range of treatment approaches for TS, but only a few are specifically tailored to each individual patient. Clinically, most treatments focus on alleviating symptoms [6, 7]. Conventional treatment is primarily based on Western medicine [8], but it is accompanied by several undesirable side effects, such as irritability, sluggishness, dizziness, weakness, and even muscle tics, that make compliance in pediatric patients difficult. Tourette’s syndrome is often a protracted and difficult-to-heal disease, and relapses can easily occur. In addition, patients are vulnerable to discrimination and the consequent psychological and emotional burden that patients and their families experience results in a significant decline in their quality of life.

In China, a growing number of parents are willing to use traditional Chinese medicine (TCM) to help their children. According to TCM theory, an internal disturbance of the liver wind and phlegm fire is the common pathogenesis of TS in children. Primary goals of treatment include calming the liver, settling the wind, clearing the heart, and soothing the nerves [9]. Herbal medicines from TCM have shown promise in the treatment of TS in clinical trials. Furthermore, a number of Chinese patent medications with improved portability and use have been developed in recent years [10].

After years of clinical use, Shaomazhijing granules (formerly known as Wuling granules) have demonstrated good curative effects [11]. However, there is scant evidence to support its efficacy and safety. Accordingly, we designed this clinical trial. In this study, we used the Yale Global Tic Severity Scale (YGTSS) [12] and the Traditional Chinese Medicine (TCM) syndrome quantitative classification scale [13] for evaluating efficacy. Zheng [14] used the YGTSS in their study but did not report the results of the TCM syndrome quantitative classification scale. (Table 1). Therefore, in this study, we have also used the results of the TCM syndrome quantitative classification scale as an indicator of the drug’s therapeutic efficacy. The details of our clinical trial are described in the following sections.

**Table 1:** Treatment outcomes measured with YGTSS in patients with TS

	Placebo (*n* = 116)		Tiapride (*n* = 123)		Shaomazhijing (*n* = 362)	
Variables	Mean ± SD	Within-group effect size	Mean ± SD	Within-group effect size	Mean ± SD	Within-group effect size
Global						
Baseline	50.0 ± 12.1		51.4 ± 11.8		52.1 ± 12.8	
Week 2	44.4 ± 14.0	0.428	43.2 ± 14.7	0.615	44.2 ± 14.6	0.575
Week 8	31.5 ± 15.2	1.347	21.2 ± 13.8	2.352	22.3 ± 15.1	2.129
TTS						
Baseline	22.7 ± 6.7		23.1 ± 6.9		23.7 ± 6.8	
Week 2	19.9 ± 7.7	0.388	19.2 ± 7.4	0.545	19.6 ± 7.3	0.581
Week 8	14.4 ± 7.5	1.167	10.1 ± 6.4	1.953	10.6 ± 6.8	1.926
Impairment						
Baseline	27.3 ± 8.0		28.3 ± 8.3		28.3 ± 8.3	
Week 2	24.6 ± 8.1	0.335	23.9 ± 8.7	0.518	24.4 ± 9.0	0.451
Week 8	17.2 ± 9.2	1.172	11.2 ± 8.1	2.073	11.6 ± 9.7	1.850
	Shaomazhijing vs. placebo		Tiapride vs. placebo		Shaomazhijing vs. Tiapride	
	Between-group effect size	*p* value	Between-group effect size	*p* value	Between-group effect size	*p* value
Global						
Week 2	.014	.897	.084	.519	.069	.508
Week 8	.608	<.001	.710	<.001	.075	.472
TTS						
Week 2	.041	.701	.093	.461	.055	.586
Week 8	.545	<.001	.618	<.001	.075	.489
Impairment						
Week 2	.023	.831	.083	.521	.057	.588
Week 8	.584	<.001	.682	<.001	.032	.756

Overall statistical significance was analyzed using a linear mixed-effect model analysis. Pairwise comparisons were further conducted between the three groups one-way analysis of variance (ANOVA). *TS* Tourette syndrome, *YGTSS* Yale Global Tic Severity Scale, *TTS* total tic score

## Materials and methods

### Overview of the study design

This study was designed as a prospective, randomized, double-blinded, placebo-controlled study and was a registered trial (Clinical trials.gov; study no. NCT01501695). The trial protocol was approved by the respective ethics committees of the institutions involved and was conducted in accordance with standard clinical practice guidelines as per the Declaration of Helsinki. We have followed the guidelines prescribed by the Consolidated Standards of Reporting Trials (CONSORT) checklist, which is the gold standard for reporting trial results.

### Settings and participants

We selected respondents from eight medical institutions in different provinces of China to avoid sample selection bias. This trial was conducted between January 2008 and November 2010. The procedures and purpose of the trial were explained in detail to patients or their legal guardians, and informed consent was obtained prior to their participation in the trial.

The inclusion criteria were as follows: (1) pediatric patients aged 5–18 years who were diagnosed with TS as per the Diagnostic and Statistical Manual of Mental Disorders (Fifth Edition) standards [15] and were classified as having liver-yang hyperactivity, liver wind, and internal phlegm-fire disturbance (Table 2); (2) patients who had stopped using other TCM and Western drugs for TS two weeks before entering the clinical trial; this was in order to avoid interference with the research results caused by other traditional Chinese and Western medicine treatments for Tourette's syndrome,; and (3) patients who had undergone comprehensive clinical and laboratory tests and the results did not indicate complications of the heart, liver, kidneys, or other major organs, and whose liver and kidney functions were normal.

**Table 2:** The diagnostic criteria of liver-yang hyperactivity with liver wind and internal disturbance of phlegm-fire

	Symptom
Main symptom	(1) Frown and wink; (2) Open mouth and grin; (3) Shake head and shrug shoulders; (4) Shake hands and kick legs; (5) Utter foul language
Secondary symptom	(1) Irritability; (2) The palms and soles fever; (3) Restlessness; (4) Dry stool; (5) Short yellow urine; (6) Red tongue; (7) Yellow coat or thin yellow greasy coat; (8) Stringy pulse or string fast pulse

Reference to Pediatrics of Traditional Chinese Medicine,includes involuntary twitching of muscles in the face, head, neck, limbs, or trunk, accompanied by abnormal vocalizations and indecent language in the throat. The main involuntary movements include squeezing eyebrows and blinking, shaking the head, shrugging, grinning, kicking, etc. The movements are frequent and powerful, accompanied by restlessness, irritability, irritability, red tongue, yellow and greasy fur, and stringy pulse. Those who have one Main symptom and three or more secondary symptoms, that is, the syndrome differentiation is liver-yang hyperactivity & liver wind and internal disturbance of phlegm-fire

The exclusion criteria were as follows: (1) patients with a definite diagnosis of hyperactivity, epilepsy, chorea, autism, and impaired intellectual ability; (2) patients with drug-induced TS; (3) patients with renal dysfunction; (4) patients who were known to be allergic to the test drug or its components; (5) patients who were participating in clinical trials involving other drugs; and (6) patients younger than 5 years or older than 18 years.

### Sample size

Based on published reports about Shaomazhijing granules [10], the current authors followed the drug registration regulations published by the State Food and Drug Administration in 2007, which state that the Phase III clinical trial group requires no less than 300 cases. Considering a dropout rate of no more than 20%, we determined a sample size of 600 participants who were randomly divided into three groups in a ratio of 3:1:1 (for the Shaomazhijing granules, tiapride, and placebo groups, respectively).

### Herbal and placebo preparation

The Shaomazhijing and placebo granules used in this study were manufactured by Tasly Pharmaceuticals Inc. (Tianjin, China) (batch no. 2007B01/2008101 for Shaomazhijing granules and 2007 C01/2007L01 for placebo granules). The placebo herbal medicine preparation has been well validated in previous studies [16, 17]. Tiapride and the placebo were manufactured by Tasly Pharmaceuticals Inc. (Jiangsu, China) (batch no. 20070502/200902051 for tiapride and 20,070,503 for the placebo). The weights and proportions of the raw materials in the Shaomazhijing granules are listed in Table 3.

**Table 3:** Formula of Shaomazhijing granules and action of individual herbal materials

English and Chinese name	Raw weight (g)	%	pharmacological properties
*Paeoniae radix alba* (Bai-Shao)	3.23	12.9	① an inhibitory effect on the central nervous system, which could relieve muscle tension, tics, and pain, and inhibit emotional hyperactivity and excitement
*Gastrodia rhizoma* (Tian-Ma)	2.35	9.4	① potential as an anticonvulsant, neuroprotective, and hypotensive, in addition to enhancing memory and regulating skeletal muscle strength
*Tribuli fructus* (Ji-Li)	2.78	11.1	① could improve depression and agitation, enhance blood circulation, and improve visual acuity
*Uncariae ramulus cum uncis* (Gou-Teng)	2.90	11.6	① sedative and anticonvulsant effects
*Ganoderma* (Ling-Zhi)	2.15	8.6	①an inhibitory effect on the nervous system②a depressurizing effect on the circulatory system③a strengthening effect on cardiac contractility④an expectorant effect on the respiratory system⑤and a tonic effect on fatigue and weakness
*Polygoni multiflori caulis*(Shou-Wu-Teng)	2.08	8.3	①sedative and hypnotic effects, which indicated an obvious synergistic effect with pentobarbital sodium
*Ziziphi spinosae semen* (Suan-Zao-Ren)	2.08	8.3	①sleepiness and significant sedation of the central nervous system, making it useful for treating night sweats
*Schisandrae chinensis fructus* (Wu-Wei-Zi)	2.08	8.3	①rich in organic acids, vitamins, flavonoids, plant sterols, and lignans, could impart strong restorative effects.②could tonify qi, strengthen the liver, and improve memory.③ could also realize observable sedative, analgesic, and muscle relaxation effects
*Gardeniae fructus* (Zhi-Zi)	2.05	8.2	①a sedative effect that could prolong sleeping time;② could also impart anticonvulsant effects, as well as cholagogic, anti-infection, and antipyretic effects
*Arisaema cum bile*(Dan-Nan-Xing)	1.90	7.6	①observable effects in treating epilepsy and sequelae of cerebrovascular disease. ② had an expectorant effect on coughing accompanied by phlegm and thin sputum
*Scutellaria radix* (Huang-Qin)	1.43	5.7	①the ability to clear heat and eliminate dampness, purge fire, and support detoxification. ②had an anti-inflammatory effect on infections caused by enteritis, dysentery, and skin damage
In total	25.00	100	

All herbal raw materials were sourced exclusively from government-authorized traditional Chinese medicine suppliers. Each herb was examined for the presence of microorganisms, heavy metals, and pesticides to ensure its compliance with Chinese government regulations. The evaluation was further supervised by senior Chinese medicine practitioners.

The herbs were decocted to obtain the dry extract [18]. The weight ratio of raw materials to the obtained product was approximately 5:1. The finished products were sealed in opaque aluminum bags, each bag containing 5 g, equivalent to 25 g of a mixture of raw herbs. The inert placebo tablets were prepared to be identical in appearance, odor, and color to the active tiapride tablets. This arm included the preparation of mock Shaomazhijing granules and tiapride tablets, which did not contain active ingredients.

### Randomization and intervention

Using the SAS statistical analysis software, we generated the random arrangement of treatment (the trial drug and control drug, respectively) received by the 600 participants to derive the treatment allocation (randomization scheme) corresponding to serial numbers 001–600. Each of the clinical centers was allocated contiguous coded drugs in subsequent order.

This trial was conducted with the collaboration of eight hospitals in China: the Affiliated Hospital of Nanjing University of Chinese Medicine, the First Affiliated Hospital of Henan University of Chinese Medicine, the First Affiliated Hospital of Zhejiang University of Chinese Medicine, the Affiliated Hospital of Liaoning University of Chinese Medicine, the First Affiliated Hospital of Tianjin University of Chinese Medicine, the Beijing Anding Hospital of Capital Medical University, the Affiliated Brain Hospital of Nanjing Medical University, and the Second Affiliated Hospital of Tianjin University of Chinese Medicine. All centers were sorted alphabetically according to their first pinyin letter, and all test centers were randomly coded. We allocated the number of participants for each study site based on the volume of patients with tic disorders who were seen and admitted to that site in the preceding three years.

Patients were assigned to treatment groups with orally administered Shaomazhijing granules plus tiapride placebo tablets, tiapride plus Shaomazhijing placebo granules, or tiapride placebo tablets plus Shaomazhijing placebo granules for 8 weeks. Patients aged 5–12 years and 13–18 years were given 3 bags (15 g of granules) and 4.5 bags (22.5 g of granules) of Shaomazhijing granules or placebo granules every day, respectively. The specified amount of granules (1 or 1.5 bags) was to be dissolved in hot water and taken three times daily. Furthermore, the patients in both age groups took 1 tablet and 2 tablets of tiapride (100 and 200 mg, respectively) or a placebo every day for the first 2 weeks, and then took 2 tablets and 4 tablets, respectively, twice daily from weeks 3–8.

### Evaluation of the treatment results

We used the TCM syndrome quantitative classification scale to evaluate the study results (Table 4). Researchers with experience in traditional Chinese medicine underwent standardized training to ensure consistency in their assessments. After completing the training workshop, raters from all study sites had an inter-rater reliability coefficient (k value) of > 0.80 on the YGTSS and the TCM syndrome quantitative classification scale.

**Table 4:** TCM syndrome quantitative classification scale

		None (point)	Occasionally (point)	Frequent (point)	Often (point)
Main symptom	Frown and wink	0	1	2	3
	Open mouth and grin	0	1	2	3
	Shake head and shrug shoulders	0	1	2	3
	Shake hands and kick legs	0	1	2	3
	Utter foul language	0	1	2	3
		None (point)		Have (point)	
Secondary symptom	Irritability	0		1	
	Palms and soles fever	0		1	
	Restlessness	0		1	
	Dry stool	0		1	
	Short yellow urine	0		1	
	Tongue nature	Light red		Red	
		0		1	
	Coating on the tongue	Thin white		Thin yellow or thin yellow and greasy	
		0		1	

Patients made five visits in total, from enrollment to the end of treatment, i.e., in weeks 1, 2, 4, 6, and 8. During each visit, the patients were instructed to check and record the medication they received and return the drug packaging for recycling during the subsequent visit. At baseline and the completion of treatment, patients underwent a general physical examination, a routine blood test, a routine urine test, and a review of liver and kidney functions; an electrocardiogram was conducted for each patient and evaluated; and their weight was checked. In cases of abnormal examination results during treatment, the patient was followed up until the test results were normal. All patients were followed up telephonically at the end of one month after the trial.

We used the following traditional Chinese medicine syndrome efficacy evaluation criteria [13]: efficacy index = (the total score of symptoms and signs before treatment – the total score of symptoms and signs after treatment)/the total score of symptoms and signs before treatment × 100%. The main symptom efficacy index = (the main symptom score before treatment – the main symptom score after treatment)/the main symptom score before treatment × 100%. The secondary symptoms efficacy index = (the secondary symptoms score before treatment – the secondary symptoms score after treatment)/the secondary symptoms score before treatment × 100%.

The observed outcomes were classified as follows [13]:

- (1) Clinically controlled: The symptoms and signs were eliminated within 8 weeks after the start of medication; the score reduction was ≥ 95%, and there was no recurrence of symptoms for more than one month.
- (2) Excellent effectiveness: Muscle tics were significantly reduced within 8 weeks, and the score reduction was ≥ 70% and < 95%.
- (3) Improved: Muscle tics improved within 8 weeks, and the score decreased by ≥ 30% but remained at < 70%.
- (4) Ineffective: No improvement or aggravation of muscle tics occurred within 8 weeks after starting the medication, and the score reduction was < 30%.

### Statistics analysis

We performed the efficacy analysis on the intention-to-treat population, which was defined as the participants who completed the baseline examination and at least one evaluation after treatment. For the descriptive statistical analysis, qualitative indicators were expressed as a percentage; quantitative indexes were expressed as mean ± standard deviation. Normally distributed quantitative data were evaluated using the t-test; non-normally distributed quantitative data were compared using the Wilcoxon rank-sum test. We used the linear mixed-effects model to compare the main treatment outcomes of the three groups of patients over time. The intra-group effect size was the difference between the mean values before and after treatment divided by the pooled standard deviation. The effect sizes of 0.20, 0.50, 0.80, and 1.30 were expressed as"small,""medium,""large,"and"very large"effects, respectively. We used the analysis of variance for comparisons between two groups and the Cochran-Mantel–Haenszel test for comparisons among the three groups; a value of *P* ≤ 0.05 was considered statistically significant.

## Results

### Patient characteristics

**Fig. 1:**
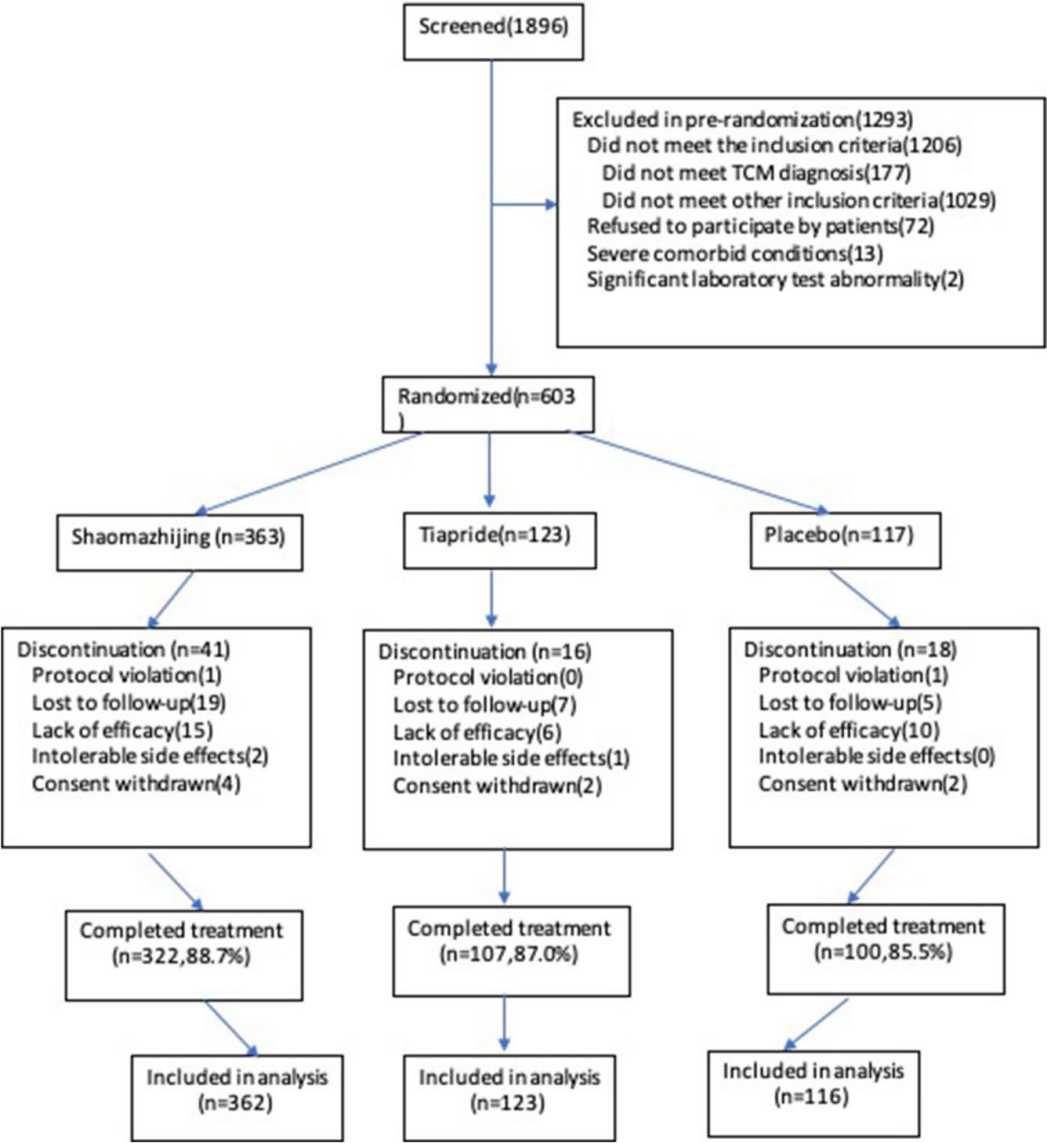
Flowchart of screening and recruitment of study subjects

A total of 603 patients were enrolled in the current trial and randomly divided into the Shaomazhijing granules (*n* = 363), tiapride (*n* = 123), and placebo (*n* = 117) groups, respectively. Subsequently, 520 patients (86.2%) completed the 8-week treatment and evaluation. Two participants who took other psychoactive drugs and herbs during the treatment period (one from the Shaomazhijing granules group and one from the placebo group) were excluded from the outcome analysis due to the violation of inclusion criteria but were included in the baseline analysis (Fig. 1). The distribution of demographic characteristics and TCM symptoms among the three groups was similar; none of the differences among the groups was statistically significant (Table 5).

**Table 5:** Baseline characteristics of patient

Detailed statement/combination statements Item	Shaomazhijing (*n* = 363)	Tiapride (*n* = 123)	Placebo (*n* = 117)	*p* value
Male, n (%)	306 (84.3)	107 (87.0)	98(83.8)	0.720
Age (years)	9.56 ± 3.03	10.02 ± 3.12	9.94 ± 2.75	0.147
Height(cm)	139.65 ± 17.57	142.00 ± 18.51	140.72 ± 16.63	0.361
Body weight(kg)	35.46 ± 13.14	38.16 ± 14.53	36.77 ± 13.06	0.171
TCM symptom	10.56 ± 2.89	10.29 ± 2.86	10.13 ± 2.71	0.306

### Treatment outcomes

The treatment outcomes are summarized in Tables 5 and 6. Linear mixed-effects model analysis showed that the three variables were significantly reduced at week 2 and week 8 compared to baseline, with the effect size of 0.383–2.444.

**Table 6:** Treatment outcomes measured with quantitative table of TS classification in patient

Variables	Shaomazhijing (*n* = 362)		Tiapride *(n* = 123)		Placebo (*n* = 116)		
	Mean ± SD	Within-group effect size	Mean ± SD	Within-group effect size	Mean ± SD	Within-group effect size	*p* value^a^
Global							
Baseline	10.56 ± 2.89		10.29 ± 2.86		10.13 ± 2.71		0.306
Week 2	7.88 ± 3.08	0.795	8.19 ± 3.20	0.568	8.11 ± 3.19	0.470	0.012
Week 8	3.85 ± 2.73	2.388	4.53 ± 2.56	2.125	5.88 ± 3.22	1.433	< 0.001
Main symptom							
Baseline	5.85 ± 2.53		5.59 ± 2.49		5.43 ± 2.35		0.214
Week 2	4.43 ± 2.46	0.597	4.25 ± 2.58	0.567	4.36 ± 2.37	0.383	0.247
Week 8	2.23 ± 1.86	1.649	2.24 ± 1.88	1.533	3.24 ± 2.35	0.932	< 0.001
Secondary symptom							
Baseline	4.71 ± 1.15		4.70 ± 1.25		4.70 ± 1.10		0.921
Week 2	3.45 ± 1.34	0.906	3.94 ± 1.35	0.429	3.75 ± 1.44	0.508	0.001
Week 8	1.63 ± 1.37	2.444	2.29 ± 1.37	1.840	2.64 ± 1.34	1.689	< 0.001

^a^ Comparison of the difference before and after treatment among the three groups

At 8 weeks, significant differences were observed among the three groups in TCM syndrome scores, primary symptom scores, and secondary symptom scores pre- and post-treatment (*P* < 0.001, Table 6). The Shaomazhijing granules and tiapride groups showed similar improvement for the primary symptom (*P* = 0.969). The Shaomazhijing granules group showed better results in the improvement of secondary symptoms compared to the tiapride group (*P* = 0.016).

The clinical control rates in the Shaomazhijing granules, tiapride, and placebo groups were 3.45%, 2.86%, and 1.04%, respectively. the clinical response in the Shaomazhijing granules, tiapride, and placebo groups were 44.51%, 26.67%, and 12.50%, respectively (*P* < 0.001, Table 7).

**Table 7:** Total curative effect of improvement of TCM symptoms of child patient in three groups after 8 weeks of treatment

Curative effect	Shaomazhijing (%)	Tiapride (%)	Placebo (%)
Clinical control	11(3.45)	3(2.86)	1(1.04)
Excellent	142(44.51)	28(26.67)	12(12.50)
Improved	142(44.51)	62(59.05)	49(51.04)
Ineffective	24(7.52)	12(11.43)	34(35.42)
Total	319	105	96
*p* value	< 0.001		

### Adverse events

**Fig. 2:**
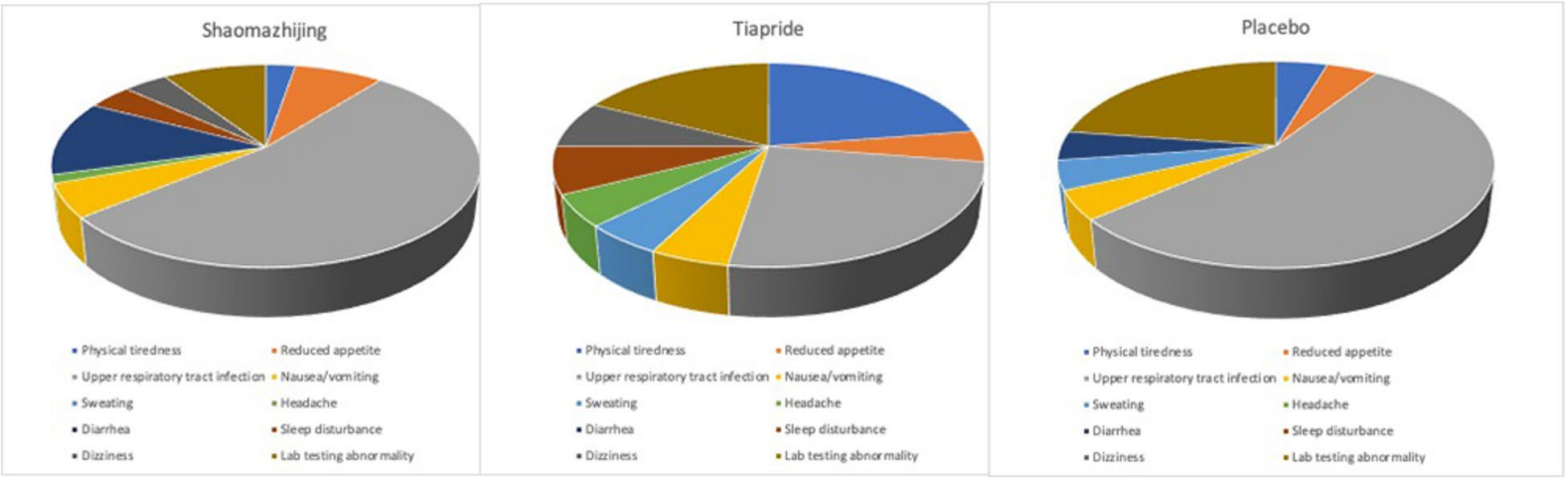
The incidence of adverse events across the three groups

The rate of adverse events across the three groups is summarized in Fig. 2. A total of 95 cases of adverse events were reported, among which 50 occurred in the Shaomazhijing granules group (13.8%), 32 in the tiapride group (26.0%), and 13 in the placebo group (11.2%). Shaomazhijing granules group had significantly less physical fatigue (0.6% vs. 7.3%, respectively), sleep disturbance (0.8% vs. 8.9%, respectively), and dizziness (0.8% vs. 4.1%, respectively) compared to the tiapride group (X^2^ ≥ 9.260, df = 2, *P* < 0.001).

## Discussion

Alternative therapies like TCM have been shown to have some benefit in the treatment of TS, although their efficacy is still debatable. TCM theory considers TS a disease related to liver wind; although the main lesion sites of this disease are in the liver, it manifests in the heart, lungs, spleen, and kidneys. The main pathological factors are liver wind and phlegm fire. Internal hyperactivity of liver wind is the main pathological feature of TS, while the main mechanism of the disease is the interaction of liver wind and phlegm fire [9]. In TCM, the clinical manifestations of excessive liver wind include blinking, shrugging, shaking the head, frowning, and limb twitching. The clinical manifestations of excessive phlegm and fire include impatience, irritability, warm hands and feet, and restlessness in sleep.

The treatment of infantile muscle tic syndrome in TCM mainly targets the liver wind and the phlegm wind. Since the liver is considered to be the source of the disease, it is also considered the root cause of TS [19]. Through long-term clinical observation, Juan found that the"heart and liver"understanding of the pathogenesis of this disease in TCM encompasses the neurological and mental functions of modern medicine, confirming the close relationship between its onset and the liver [20]. Research by Wang has shown that the understanding of the involvement of the liver as per TCM theory is related to the neuroendocrine immune network, which is at the core of regulating the psychological stress response of the human body [21]. A meta-analysis of the 47 trials that utilized YGTSS to assess tic severity found that those who used TCM showed significant improvement in YGTSS-Total Tic Scores (YGTSS-TTS) compared to those in the control groups (SMD = −0:21, 95%CI: −0.29 to −0.14, I2 = 12:30%) [22], but it did not include the TCM characteristics of Tourette's syndrome.

**Fig. 3:**
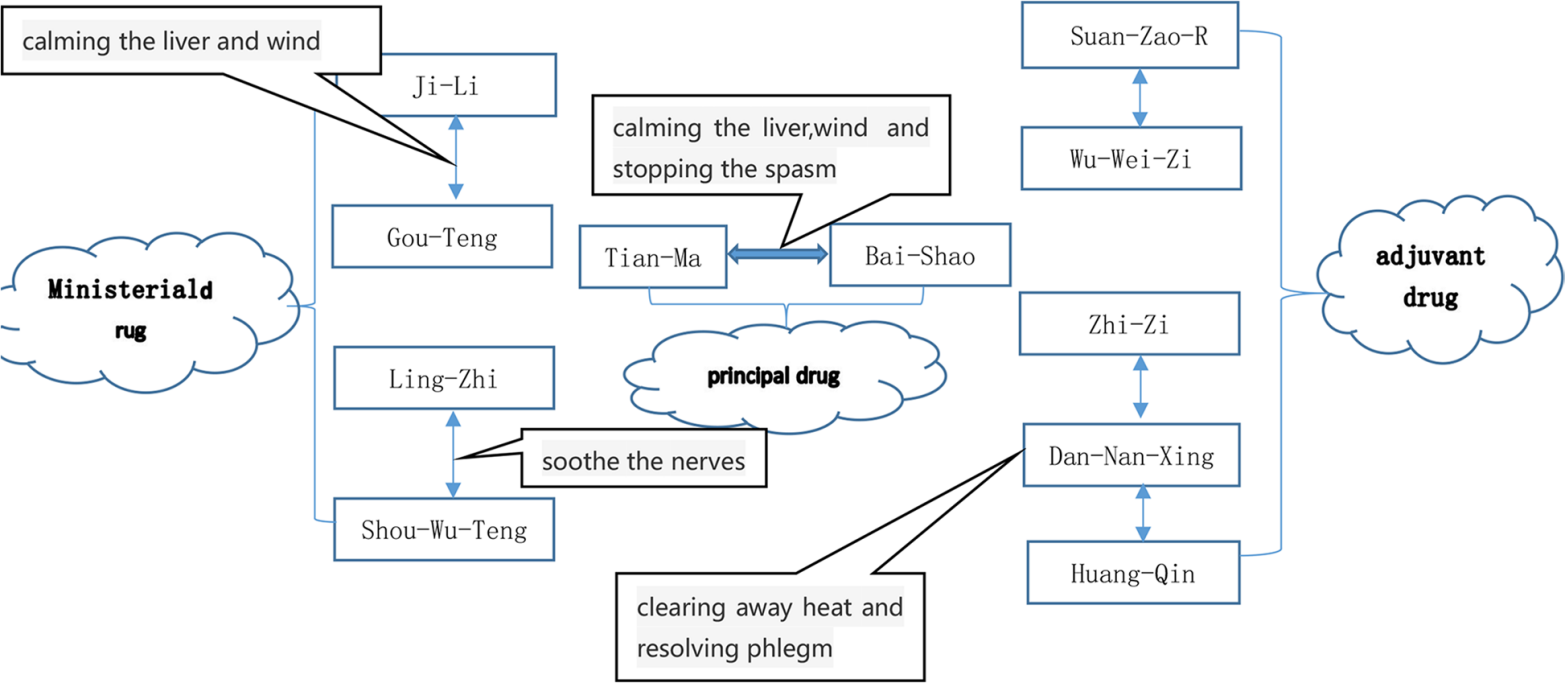
Schematic diagram of prescription analysis of Shaomazhijing granules

Shaomazhijing granules focus on the liver for treating the muscle tics associated with TS, following the principles of calming liver wind, clearing heat, and reducing phlegm. This is a Chinese patent medicine comprising 11 herbs (*Paeoniae radix alba*, *Gastrodia rhizoma*, *Tribuli fructus*, *Uncariae ramulus cum uncis*, *Ganoderma*, *Polygoni multiflori caulis*, *Ziziphi spinosae semen*, *Schisandrae chinensis fructus*, *Gardeniae fructus*, *Arisaema cum bile*, and *Scutellaria radix*) that has the effects of suppressing liver yang, extinguishing wind and muscle tics, clearing fire, and eliminating phlegm (Fig. 3). When prescribing treatment for TS, *Paeoniae radix alba* and *Gastrodia rhizoma* are the dominant drugs, and their combination can calm the liver wind and stop muscle tics. *Tribuli fructus, Uncariae ramulus cum uncis, Ganoderma,* and *Polygoni multiflori caulis* are ministerial drugs, the combination of which can calm the liver, clear heat, and subdue yang. *Ziziphi spinosae semen, Schisandrae chinensis fructus, Gardeniae fructus, Arisaema cum bile,* and *Scutellaria radix* are the adjuvant drugs, and when used in combination, they can clear heat, reduce phlegm, and calm nerves.

At present, the abnormal increase of dopamine activity, dopamine receptor hypersensitivity, norepinephrine dysfunction, or abnormal 5-hydroxytryptamine level in the nervous system are all thought to be closely related to the onset of Tourette's syndrome [23]. Some cases of tic disorder are related to autoimmune damage after infection, with about 10% being related to Group A beta hemolytic streptococcus (GABHS) infection [4]. This suggests that the efficacy of Shaomazhijing observed in this study may be related to its multiple pharmacological and therapeutic properties. In a study, it was found that in the Shaomazhijing formulation, Geissoschizine methyl ether (GM) present in *Uncaria* stems and hooks and 18β glycyrrhetinic acid present in *Glycyrrhiza* could be absorbed into the bloodstream and crossed the blood–brain barrier, reaching the frontal cortex and hippocampus in rats, and GM exerted a partial agonist effect on D2 receptors of dopamine [24].

The anti-inflammatory effects of the *Radix Scutellariae* were attributed to its ability to inhibit the NF-κB pathway via the activation of the PPARγ/RXR pathway, thereby reducing the expression of inflammation-related cytokines [25]. *Baicalin* ameliorated neuroinflammation-induced depressive-like behaviors through the inhibition of TLR4 expression via the PI3 K/AKT/FoxO1 pathway [26]. *Radix Scutellariae* could ameliorate depressive-like behaviors, increase the expression of the antiapoptotic protein B-cell lymphoma 2 (BCL-2), reduce the expression of the proapoptotic protein BCL-2-associated X (BAX), and increase the number of doublecortin- (DCX-), microtubule-associated protein 2- (MAP2-), and neuronal nucleus- (NeuN-) positive cells in the hippocampus [27]. Recent research has indicated that *Fructus Schisandrae Chinensis* and its active ingredients play a protective role in neurological diseases, including cerebrovascular diseases, neurodegenerative diseases, and depression. The key neuroprotective mechanisms of *FSC* and its active ingredients have been demonstrated to include antioxidation, suppression of apoptosis, anti-inflammation, regulation of neurotransmitters, and modulation of brain-derived neurotrophic factor (BDNF)-related pathways [28].

The current large-scale placebo-controlled study evaluated the efficacy and safety of TCM as monotherapy for TS patients for the first time. We evaluated the treatment outcomes using the TCM syndrome quantitative classification scale and the YGTSS. The primary symptom was muscle tics, while secondary symptoms included changes in moods and emotions and psychological effects. At week 8 of treatment, the clinical control rate and clinical effectiveness rate of TCM syndromes in the Shaomazhijing granules group were higher than in the tiapride and placebo groups; the pre- and post-treatment differences in TCM primary and secondary symptom scores among the three groups were statistically significant (*P* < 0.001). Improvement of the primary symptom (muscle tics) was comparable between the Shaomazhijing granules and tiapride groups (*P* = 0.969). Secondary symptoms improved more in the Shaomazhijing granules group when compared to the tiapride and placebo groups (*P* < 0.05), suggesting that Shaomazhijing granules may be helpful in treating TCM syndromes among pediatric patients. The primary reason for this outcome was that some of the drugs included in the treatment formula improved the mood of the patients and their psychological state.

Compared with the tiapride group, the incidence of overall adverse events in the Shaomazhijing granules group was significantly lower, particularly in terms of physical fatigue, sleep disturbances, and dizziness. Previous studies on the use of herbal medicine as an additional therapy in patients with mood disorders found similar outcomes [17, 29, 30]. These effects were related to the pharmacological characteristics of the active ingredients in the Shaomazhijing granules. *Ganoderma* and *Polygoni multiflori caulis* are often used to alleviate fatigue; *Paeoniae radix alba*, *Gastrodia rhizoma*, *Tribuli fructus*, and *Uncariae ramulus cum uncis* are used to treat hypertensive vertigo and tremors; and *Polygoni multiflori caulis*, *Ziziphi spinosae semen*, *Schisandrae chinensis fructus*, and *Gardeniae fructus* are used to treat insomnia and agitation [31]. In the present study, we also found that the biochemistry of the three groups did not significantly differ, suggesting that the oral administration of Shaomazhijing granules for 8 weeks was safe.

There are some limitations to this study. First, we did not factor the symptoms of comorbidities into our data analysis. TS is often associated with attention-deficit/hyperactivity disorders (ADHD) and obsessive–compulsive disorders. Further research is required to evaluate whether Shaomazhijing granules are effective in treating additional neurodevelopmental disorders. Second, most patients with TS required long-term treatment; we did not examine the follow-ups and long-term results in this study, and, as such, more research is required to evaluate the long-term safety and efficacy of Shaomazhijing granules.

In conclusion, our findings suggest that the clinical efficacy of Shaomazhijing granules in the treatment of Tourette's syndrome was comparable to that of tiapride in reducing the primary symptoms (muscle tics) of TS. Besides, it showed better efficacy in improving secondary (emotional and psychological) symptoms. Its safety profile is better than tiapride. Thus, the use of Shaomazhijing granules shows promising therapeutic application prospects in the treatment of TS.

## Data Availability

All data generated or analysed during this study are included in this article. Further enquiries can be directed to the corresponding author.
